# FBXW7: a critical tumor suppressor of human cancers

**DOI:** 10.1186/s12943-018-0857-2

**Published:** 2018-08-07

**Authors:** Chien-Hung Yeh, Marcia Bellon, Christophe Nicot

**Affiliations:** Department of Pathology and Laboratory Medicine, Center for Viral Pathogenesis, University of Kansas Medical Center, 3901 Rainbow Boulevard, Kansas City, KS 66160 USA

**Keywords:** FBXW7, CDC4, Cancer, C-myc, Cyclin E, mcl-1, Notch, HTLV, mTOR, Jun

## Abstract

The ubiquitin-proteasome system (UPS) is involved in multiple aspects of cellular processes, such as cell cycle progression, cellular differentiation, and survival (Davis RJ et al., Cancer Cell 26:455-64, 2014; Skaar JR et al., Nat Rev Drug Discov 13:889-903, 2014; Nakayama KI and Nakayama K, Nat Rev Cancer 6:369-81, 2006). F-box and WD repeat domain containing 7 (FBXW7), also known as Sel10, hCDC4 or hAgo, is a member of the F-box protein family, which functions as the substrate recognition component of the SCF E3 ubiquitin ligase. FBXW7 is a critical tumor suppressor and one of the most commonly deregulated ubiquitin-proteasome system proteins in human cancer. FBXW7 controls proteasome-mediated degradation of oncoproteins such as cyclin E, c-Myc, Mcl-1, mTOR, Jun, Notch and AURKA. Consistent with the tumor suppressor role of FBXW7, it is located at chromosome 4q32, a genomic region deleted in more than 30% of all human cancers (Spruck CH et al., Cancer Res 62:4535-9, 2002). Genetic profiles of human cancers based on high-throughput sequencing have revealed that FBXW7 is frequently mutated in human cancers. In addition to genetic mutations, other mechanisms involving microRNA, long non-coding RNA, and specific oncogenic signaling pathways can inactivate FBXW7 functions in cancer cells. In the following sections, we will discuss the regulation of FBXW7, its role in oncogenesis, and the clinical implications and prognostic value of loss of function of FBXW7 in human cancers.

## FBXW7 oncogenic substrates and its role as a tumor suppressor in cancer

**Fig. 1:**
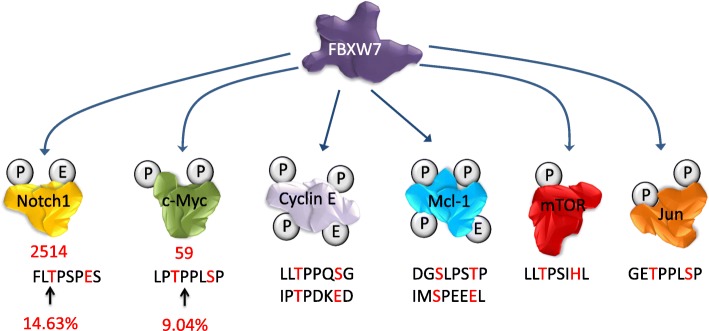
FBXW7 substrates and their conserved CDC4 phosphodegron. FBXW7 causes proteasome-mediated degradation of Notch1, c-Myc, cyclin E, Mcl-1, mTOR, and Jun. The amino acid sequence under each FBXW7 substrate indicates the conserved CDC4 phosphodegron (the phosphorylation residue “0” and “+ 4” positions are highlighted in red). The amino acid number and percentage indicate the hotspot mutations in human cancers. Mutation percentage data were retrieved from the COSMIC database

FBXW7 is a member of the F-box protein family, which is part of the Skp1-Cdc53/Cullin-F-box-protein complex (SCF/β-TrCP). The SCF complex is an E3-ubiquitin ligase that ubiquinates proteins and triggers proteasome degradation. The SCF complex is composed of four subunits – Skp1, Cul-1 (Cullin/Cdc53), and Rbx1 proteins – and a fourth component consisting of a variable F-box protein that determines substrate specificity. These F-box proteins, including FBXW7, are responsible for recognizing and binding to phosphorylated substrates regulating their turnover. In mammalian cells there are three FBXW7 isoforms: FBXW7α, FBXW7β, and FBXW7γ, which differ in their 5’-UTR and N-terminal coding regions. These isoforms have distinct cellular localizations restricting interactions with specific partners and their functions. FBXW7α is localized in the nucleoplasm, FBXW7β is cytoplasmic, and FBXW7γ is nucleolar [1]. FBXW7α is ubiquitously expressed in the majority of proliferating cells and performs most of the known FBXW7 functions. All FBXW7 isoforms recognize their substrates through the presence of a conserved CDC4 phosphodegron (CPD) motif, which requires the substrate to be phosphorylated at specific residues in order to be ubiquitinated and targeted for proteasome degradation [2–4] (Fig. 1).

FBXW7 is classified as a tumor suppressor gene. FBXW7 expression increases following p53 activation, causes tumorigenesis in wild-type p53/FBXW7 mutated mice, and often demonstrates mutational and allelic loss in various human cancers [5]. Although FBXW7 −/− mice are embryonic lethal, loss of FBXW7 inhibits cell division, stem cell differentiation, enhances chromosomal instability, and can cause cancer in hematopoietic cells [6–9]. The majority of FBXW7 targets are known as proto-oncogenes, further supporting FBXW7’s role as a tumor suppressor. Using FBXW7 knock-outs, it has been suggested that there may be close to 90 FBXW7 substrates [10]. The level of FBXW7-mediated degradation can be regulated two-fold [11]. FBXW7 binding can be reinforced through multiple phosphorylation sites within the CPD motif of the substrate, allowing for additional cellular pathways to converge and regulate substrate phosphorylation/degradation. Additionally, dimerization of the FBXW7 protein itself can increase the activity and the binding of FBXW7 to substrates, especially for those substrates with weak phosphodegrons. Glycogen synthase kinase 3 beta (GSK3β) is commonly recognized as a significant player in phosphorylating those substrates destined for FBXW7-mediated degradation [12]. Some of the most cancer-relevant substrates targeted by FBXW7 are discussed below (Fig. 1).

### c-MYC

c-Myc is a master regulator of cellular gene transcription and is reported to bind to up to 15% of promoters in the human genome [13]. c-Myc affects cellular proliferation, survival pathways, and is frequently deregulated in human tumors [14]. Due to its central role in regulating cellular pathways, c-Myc expression and activity are tightly regulated. Among the proteins that can post-transcriptionally regulate c-Myc turnover are Skp2 and FBXW7. Studies have shown that knock-down of FBXW7 increases c-Myc expression and its activity, and FBXW7 −/− mouse embryonic stem cells have elevated c-Myc expression levels [15]. FBXW7 regulates leukemia-initiating cell (LIC) homeostasis [16] by controlling c-Myc levels, which correlates precisely with LIC activity. Loss of FBXW7 results in c-Myc accumulation and p53-dependent apoptosis, preventing initiation of Bcr-Abl-induced chronic myelogenous leukemia (CML) and B-cell acute lymphoblastic leukemia (B-ALL) [17]. The phosphorylation of residues Thr58 and Ser62 within the c-Myc CPD is an essential step for FBXW7-mediated degradation and is associated with reduced tumor cell proliferation and survival [1, 12, 15]. However, specific mutations at Thr58 and Ser62 that can prevent c-Myc degradation by FBXW7 are found in many cancers [12, 15, 18]. In fact, a c-Myc mutation at residue Thr58 is the most frequently mutated c-Myc site in human B-cell lymphomas [18]. Unlike the homotypic poly-ubiquitination that occurs with FBXW7-mediated c-Myc degradation, SCF, along with the Ubiquitin Conjugating Enzyme E2 D1 (UbcH5), can add heterotypic poly-ubiquitination chains to the N-terminal of c-Myc. This action antagonizes FBXW7-mediated degradation and stabilizes c-Myc protein levels [19]. Therefore, different poly-ubiquitination chains determine different fates of the c-Myc protein. Finally, ubiquitin-specific proteases (USP) Usp28 and Usp36 have also been shown to counteract FBXW7-mediated c-Myc degradation by deubiquitinating c-Myc [20, 21].

### Cyclin E

Cyclin E/cyclin-dependent kinase Cdk2 (cyclin E/CDK2) regulates cell cycle progression by licensing DNA replication at the G1-S phase transition [22]. Abnormal cyclin E expression is observed in many cancers [23–25]. Mutations of FBXW7 that impair its ability to degrade cyclin E have been reported to result in chromosome instability, aneuploidy, and to play a role in tumorigenesis [23, 24, 26, 27]. In FBXW7 knock-out mice, elevated levels of cyclin E are correlated with an increased rate of DNA synthesis [28]. During normal cell cycle, cyclin E amounts need to be regulated in order to reduce and limit the amount of cyclin E in the cell. The phosphorylation of cyclin E by cyclin dependent kinase 2 (CDK2) and GSK3β, at Ser384 and Thr380, respectively, are essential for FBXW7-mediated degradation [2, 4]. During interphase of the cell cycle, protein phosphatases, such as PP2A-B56, have been shown to dephosphorylate Ser384, leading to reduced FBXW7 degradation and higher levels of cyclin E [29]. Additionally, phosphatase PP2A-B55 can dephosphorylate the N- and the C-terminal phosphodegrons of cyclin E within an active CDK2 complex [30]. This action opposes FBXW7-mediated degradation and prevents autocatalytic degradation of cyclin E. An oncogenic active Ras signaling pathway has been shown to inhibit FBXW7-mediated cyclin E degradation by activating the mitogen-activated protein kinase (MAPK) pathway [31]. Mechanically, activated Ras impaired the interaction between FBXW7 and cyclin E, and hence reduced cyclin E ubiquitination [31].

### Notch1

Notch ((Drosophila) Homolog 1 Translocation-Associated) proteins are a family of receptors responsible for activation of Notch signaling pathways. The Notch pathway has numerous cellular effects, including roles in development, cellular differentiation, regulation of stem cells, cellular proliferation, and cell death [32]. Physiological activation of Notch involves interactions between the Notch receptor and a ligand of the Delta/Serrate/LAG-2 family. This triggers a series of proteolytic cleavages within Notch that release the nuclear intracellular domain (NICD). NICD then translocates to the nucleus to activate transcription of target genes [33]. Notch de-regulation has been linked to numerous cancers and inheritable, cardiovascular, and cerebrovascular diseases [34]. Termination of Notch signaling requires phosphorylation of NICD in the PEST domain through the cyclin C/CDK8 complex or through cyclin C complexing with CDK19 or CDK3 [35, 36]. These interactions cause phosphorylation at residues Thr2512, Ser2514, and Ser2517. In addition, GSK3α/β and ILK (Integrin-linked kinase) also phosphorylate NICD for FBXW7-mediated degradation [37–39]. This event is mediated by GSK3α/β phosphorylation at Thr1851, Thr2123, and Thr2125 and ILK phosphorylation at Ser2173. Genetic mutations of NICD preventing FBXW7-mediated turnover have been demonstrated in many hematopoietic and solid tumors [40, 41]. In addition to the Notch1 mutations, more than 30% of pediatric T-ALL patients harbor FBXW7 mutations [42]. These mutations have been shown to disrupt the interaction between FBXW7 and NICD [42, 43]. Importantly, T-ALL cells carrying FBXW7 mutations demonstrate an extended NICD protein half-life and are generally resistant to the Notch inhibitor, MRK-003 GSI [42, 43].

### MCL-1

Myeloid cell leukemia-1 (Mcl-1) belongs to the BCL-2 family and regulates apoptosis in normal and cancer cells [44]. Mcl-1 amplification is a common genetic abnormality observed in human cancer [45]. Mcl-1 has a very short half-life and its expression is tightly regulated. FBXW7 interacts with and targets Mcl-1 for degradation following the phosphorylation of Ser159 and Thr163 present in the CPD of Mcl-1 [46, 47]. These two sites represent the major phosphorylation targets for GSK3, with Ser121 serving as an additional minor phosphorylation site. Studies have shown that an AKT inhibitor (API-1), which is upstream of GSK3, requires both FBXW7-dependent and FBXW7-independent (through β-TrCP) targeting in order to mediate full degradation of Mcl-1 [46]. In addition, mTOR complex 2 (mTOR2) has been shown to inhibit GSK3-dependent FBXW7-mediated degradation of Mcl-1 [48]. This event leads to stabilization and increased levels of Mcl-1. In human T-ALL, loss of FBXW7 is associated with Mcl-1 overexpression and increased susceptibility to the multi-kinase inhibitor Sorafenib, and increased resistance to the BCL2 antagonist ABT-737 [47]. Furthermore, mutation of FBXW7 in squamous cell carcinoma (SCC) has been shown to increase Mcl-1 expression and promote resistance to standard chemotherapy [49].

### mTOR

The mammalian target of rapamycin (mTOR) is a serine/threonine kinase and member of the phosphoinositide 3-kinase-related kinase (PIKK) family, and plays important roles in cell proliferation, metabolism, survival, and autophagy. mTOR resides in a protein complex that contains either mTORC1 or mTORC2. mTOR turnover is regulated by FBXW7-mediated ubiquitination and degradation [50]. FBXW7 knock-outs show loss of mTOR, p-mTOR, and S6-kinase (p-SK6), a known downstream target. RICTOR (Rapamycin-insensitive companion of mammalian target of rapamycin), a component of mTOR2, can also be regulated by FBXW7 [51]. This event is dependent upon GSK3 phosphorylation of RICTOR at residue Thr1695 [51]. In contrast, activation of AKT inhibits GSK3 activity, thus up-regulating RICTOR expression and mTORC2 activity. This suggests that FBXW7 may act as a switch to regulate mTORC2/ AKT activity [51]. Radiation-induced loss of mTOR is associated with activation of FBXW7 expression, resulting in FBXW7-dependent degradation of mTOR [52]. Furthermore, rapamycin treatment delays tumor development in FBXW7/p53 double heterozygous (FBXW7+/−; p53+/−) mice, but not p53 single heterozygous (p53+/−) mice, after exposure to radiation [52]. These observations suggest the importance of mTOR and FBXW7 in radiation-induced tumor formation. Moreover, increased mTOR expression in FBXW7 deleted or mutated cells sensitizes these cells to rapamycin treatment [50]. Therefore, inactivation of FBXW7 may be used as a biomarker to identify patients that may respond better to rapamycin treatment [50].

### Jun

Activator protein 1 (AP1) dimers are mainly composed of Jun-Fos and Jun-ATF2 proteins, important factors in controlling cell proliferation, stress responses, and apoptosis. Under physiological conditions Jun expression is controlled by FBXW7-mediated ubiquitination and proteasome degradation. Mechanistically, FBXW7 requires GSK3β-mediated C-terminal phosphorylation of c-Jun at Thr239 and Ser243 [53]. In contrast, the phosphorylation at the N-terminus of c-Jun by Jun N-terminal kinases (JNKs) significantly increases its half-life. Further studies showed that the receptor for activated C-kinase 1 (Rack1) can form a complex with c-Jun and FBXW7, but only recruits C-terminal phosphorylated c-Jun. Hence, c-Jun phosphorylated at its N-terminus is excluded from the FBXW7-Rack1 complex and protected from ubiquitination and degradation. The importance of FBXW7 regulation of Jun is further demonstrated in gut-specific (Villin-Cre) FBXW7 knock-out mice. These mice develop adenoma at 9–10 months of age and present elevated c-Jun expression [54]. In contrast, ectopic expression of FBXW7 in renal cell carcinoma (RCC) cells reduces c-Jun expression and leads to reduced cellular proliferation and apoptosis [55].

### Aurora kinase A (AURKA)

AURKA is a serine/threonine kinase playing an essential role in the regulation of the mitotic checkpoint, spindle assembly checkpoint (SAC), centrosome duplication, and cytokinesis. BUB and MAD proteins inhibit the ubiquitin ligase activity of the Anaphase Promoting Complex/Cyclosome (APC/C) during mitosis to ensure cells with unaligned chromosomes do not prematurely enter anaphase. In contrast, FBXW7 controls the E3 ligase activity of APC Cdh1 by modulating the expression of cyclin E. Thus, in FBXW7-deficient cells, increased cyclin E expression inactivates APC Cdh1 via direct phosphorylation of Cdh1. Increased expression of AURKA is associated with aneuploidy, which may trigger apoptosis or cellular transformation. AURKA is located on chromosome 20q13.2, a region commonly amplified in cancer. Studies have demonstrated that loss of phosphatase and tensin homologue deleted on chromosome 10 (PTEN) results in an increased half-life of AURKA by reducing FBXW7-dependent degradation of AURKA, leading to genetic instability [56, 57]. Further studies identified that Thr217 and Glu221 amino acids were indispensable for direct interaction between AURKA and FBXW7 and that Ser245 and Ser387 phosphorylation by GSK3β was required for FBXW7-mediated proteasome degradation of AURKA [58]. Overexpression of FBXW7 reduces expression of AURKA in ovarian cancer cells [59]. In addition, radiation-induced tumor latency is shorter in FBXW7 heterozygous and PTEN heterozygous knock-out mice (FBXW7+/−; PTEN+/−) compared to FBXW7 heterozygous knock-out mice (FBXW7+/−) and PTEN heterozygous knock-out mice (PTEN+/−) [58]. Therefore, FBXW7 can cooperate with PTEN to target AURKA and suppress tumorigenesis [58].

### Oncogenic activities of FBXW7

The role of FBXW7 as a tumor suppressor is well established. However, novel oncogenic FBXW7 mutants recently identified in human T-cell leukemia virus (HTLV-I) transformed adult T-cell leukemia (ATL) cells suggest a possible oncogenic role for FBXW7. FBXW7 mutations D510E and D527G demonstrate transforming activity when co-expressed with either HTLV-I’s oncoprotein Tax, mutated p53 (R276H), or mutated c-Myc (F138C) [60]. These observations are relevant since these mutations in p53 and c-Myc have been previously detected in a variety of human cancers [60]. The transforming activity of FBXW7 mutants was further demonstrated by their ability to provide IL-2-independent growth of Tax-immortalized human T cells and by increasing tumor formation in a xenograft mouse model of ATL [60]. These data suggest that FBXW7, normally a tumor suppressor, may act as an oncogene under specific circumstances. Other reports have also described oncogenic activity for FBXW7 [61]. In epidermal cells, loss of presenilin (PS), which is a catalytic component of gamma-secretase, leads to up-regulation of FBXW7 and subsequent stabilization of epidermal growth factor receptor (EGFR) [61]. Recently, FBXW7 was shown to be recruited to radiation-induced DNA damage sites upon phosphorylation by ATM Serine/Threonine Kinase (ATM) [62]. FBXW7 promoted K63 ubiquitination of the DNA repair protein X-Ray Repair Cross Complementing 4 (XRCC4) at K296, thereby enhancing the binding of XRCC4 to the Ku70/80 heterodimer and facilitating non-homologous end joining (NHEJ) repair of double DNA strand breaks (DSBs) [62]. NHEJ is often error-prone and results in the loss of nucleotides following repair. This can then lead to increases in DNA damage and genomic instability. While these events increase resistance of cells to radiation, it may also allow survival and accumulation of genetic defects in cells that should enter apoptosis and this could ultimately culminate in cellular transformation.

## Inactivation of FBXW7 functions

### Regulation of FBXW7 expression, ubiquitination, and degradation:

The expression of FBXW7 mRNA and protein is detectable in all tested human tissues [63]. FBXW7 protein expression is high in many normal human tissues, with no tissue showing low expression of the FBXW7 protein [63]. Examination of FBXW7 protein expression by immunostaining shows that compared with normal primary tissues, FBXW7 protein expression is greatly reduced in carcinoid, glioma, liver, lung, melanoma, pancreatic, prostate, renal, skin, testis, thyroid and urothelial cancers [63]. FBXW7 expression and activity can be regulated through genetic mutations, RNA silencing, post-transcriptional mechanisms, and homodimerization. In addition to miRNA and lncRNA, protein deregulation can affect FBXW7 gene expression. For example, Hes5, a downstream target of Notch, has been shown to inhibit FBXW7β promoter gene expression, forming a feedback loop between Notch, Hes5, and FBXW7.

### Non-coding microRNA (miRNA) and long non-coding RNA (lncRNA) regulation of FBXW7 gene expression:

miRNAs can bind to the 3′ untranslated region (UTR) of a gene and target the mRNA for degradation and prevent protein translation. The miRNA miR-548 has been shown to bind directly to the 3’UTR of FBXW7 and reduce the levels of FBXW7 mRNA and protein [64]. In addition, the lncRNA, lncRNA-MIF, has been shown to counteract the action of miR-548 by acting as a miR-548 sponge [64]. Overexpression of lncRNA-MIF can therefore increase the mRNA and protein expression of FBXW7 and inhibit the expression of FBXW7 targets such as c-Myc or c-Jun. In the same manner, the lncRNAs TINCR, CASC2, and MALAT1 can also act as sponges to block inhibition of FBXW7 by miR-544a, miR-367, and miR-155, respectively [65–67]. Additional miRNAs that have been shown to bind and inhibit the 3’UTR of FBXW7 include miR-223, miR-25, miR-27, miR-32, miR-92, miR-155-3p, miR-182, and miR-503 [68–73]. The majority of these miRNAs inhibited FBXW7 downstream targets, such as cyclin E, Mcl-1, c-Jun, and c-Myc, and correlated with FBXW7 expression in various tumorigenic patient samples. In several of these cases, the correlation between a single miRNA and the gene expression of FBXW7 in patient samples was relatively weak, suggesting that more than one single miRNA is required or additional mechanisms exist to regulate FBXW7 gene expression.

### Epigenetic regulation of FBXW7 expression

FBXW7α is ubiquitously expressed across multiple cell lines and patient samples and has broad tissue distribution. In contrast, FBXW7β shows differential expression in cell lines and even in tissue localization. The FBXW7β promoter can be epigenetically regulated through DNA and histone modifications, and is methylated in 43% of a variety of cancer cell lines and 51% of primary breast cancer tumors [74]. Methylation correlated with a decrease in FBXW7β gene expression. Unexpectedly, methylation of FBXW7 is associated with a longer overall survival period in lymph node-positive breast cancer patients, although it is also associated with high-grade tumors [74]. Samples from ovarian cancer patients show decreased FBXW7 expression in patients with mutated p53 [75]. Mutations in p53 are associated with hypermethylation of the FBXW7 promoter, resulting in decreased FBXW7 gene levels. This may result from p53’s ability to increase expression of the DNA methyltransferase 1 (DNMT1). In addition to DNA modifications, histone modifications are reported to control FBXW7 expression. Enhancer of zeste homolog 2 polycomb repressive complex 2 (EZH2) is a histone methyltransferase involved in epigenetic silencing of genes. EZH2 causes the addition of three methyl groups onto the histone H3 residue, H3K27me3, of FBXW7 [76]. This leads to silencing of FBXW7 gene function, which in turn activates the Notch signaling pathway [76]. Interestingly, EZH2 has been reported to be a substrate of FBXW7 in pancreatic cancer cells and is negatively correlated with FBXW7 expression in human pancreatic cancer samples [77].

### Genetic regulation of FBXW7

**Fig. 2:**
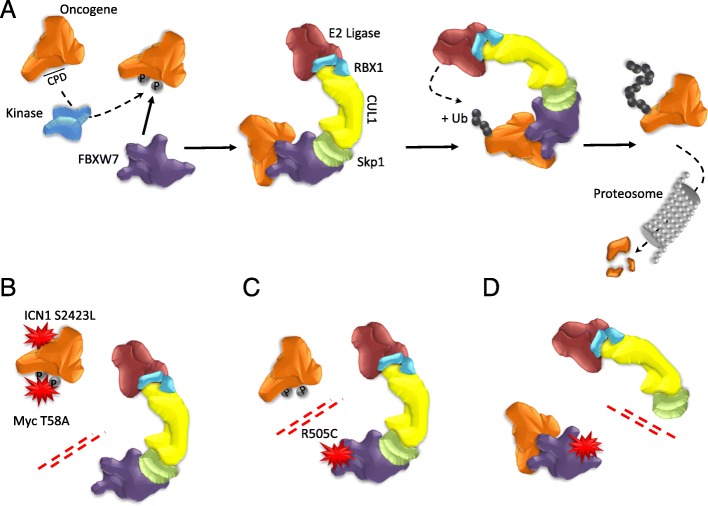
Genetic mutations impair FBXW7-mediated oncogene degradation. (**a**) FBXW7 recognizes its substrate through a conserved CDC4 phosphodegron (CPD) motif, which requires the substrate to be phosphorylated by a kinase. The SCF complex, which consists of SKP1, CUL1, RBX1 and FBXW7, interacts with the substrate through FBXW7 and adds ubiquitin (Ub) to the substrates. Poly-ubiquitinated substrates are then targeted by the proteasome for degradation. (**b**-**d**) The FBXW7-substrate interaction can be de-stabilized through: mutations in the substrate that prevent the interaction with FBXW7 (**b**), mutations in the WD40 domain of FBXW7 that impair its ability to interact with the substrate (**c**), and mutations in the FBXW7 F-box domain that inhibit its ability to interact with the substrate (**d**). These mutations have been reported in human cancers and may impair the formation of the SCF complex and stabilize FBXW7 substrates

**Fig. 3:**
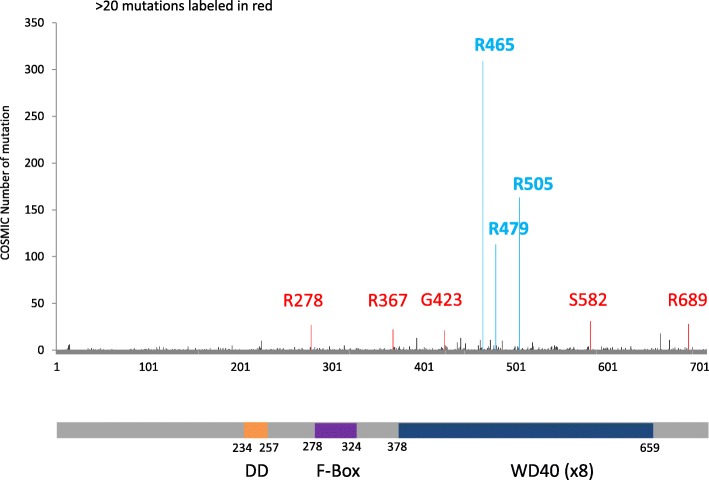
The distribution of FBXW7 mutations in the FBXW7 genome. The numbers of FBXW7 genetic alternations are retrieved from the COSMIC database. FBXW7 residues with a mutation number higher than 20 are highlighted in red and 3 FBXW7 hotspot mutations are labeled in blue. The bottom panel indicates FBXW7 protein domains (DD: dimerization domain amino acid 234–257, F-Box-like domain: amino acid 281–325 and WD40: amino acid 374–650)

**Fig. 4:**
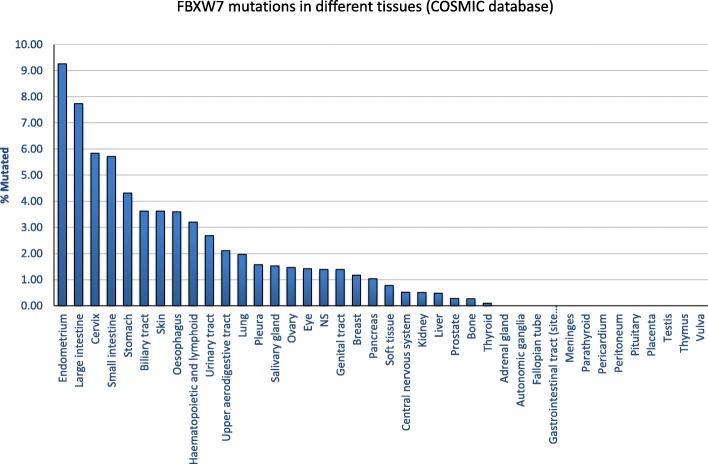
The distribution of FBXW7 mutations in different human tissues. The percentage of FBXW7 mutations varies from tissue to tissue. The endometrium has the highest percentage of FBXW7 mutations (9.26%). In contrast, no FBXW7 mutation is found in the adrenal gland, autonomic ganglia, fallopian tube, gastrointestinal tract (site indeterminate), meninges, parathyroid, pericardium, peritoneum, pituitary, placenta, testis, thymus and vulva tissues

Analyses of the Catalogue of Somatic Mutations in Cancer (COSMIC) database revealed that FBXW7 has the highest mutational frequency among all F-box and WD repeat domain containing family members (Table 1) and SCF ubiquitin ligase complexes (Table 2). FBXW7 mutations may reduce the protein’s ability to form SCF complexes and/or alter the complex conformation leading to a non-functional complex. FBXW7 interacts with SKP1 through the F-box-like domain and mutations of FBXW7 in the F-box-like domain have been reported in the COSMIC database (Fig. 2). Whether FBXW7 mutations in the F-box domain may also impair interactions with SKP1 needs to be further investigated. We performed COSMIC database meta-analyses and found an overall FBXW7 mutation rate of 2.54% across all human tumors (*n* = 1216/ *n* = 47,844) [78]. Among these, 72.70% (884/1216) of FBXW7 mutations are substitution missense mutations and 13.82% (168/1216) are substitution nonsense mutations. Insertion/deletion mutations represent 7.89% of FBXW7 mutations. Further analyses of tissue distribution of FBXW7 mutations revealed that the endometrium (85/918: 9.26%), large intestine (346/4512: 7.73%), cervix (24/411: 5.84%), small intestine (8/143: 5.59%) and stomach (51/1182: 4.31%) are the top five tumor tissues harboring FBXW7 point mutations (Fig. 3). The vast majority of FBXW7 mutations detected in cancer cells are single nucleotide changes, predominantly missense substitutions. The most common mutations are found in mutational hotspots, R505, R465 and R479, codons located in the substrate binding domain also known as tryptophan-aspartic acid motif (WD40) and represent 25.41% (309/1216), 9.29% (113/1216) and 13.40% (163/1216), respectively, of all FBXW7 mutations (Fig. 4) [78]. In addition, residues R278, R367, G423, S582 and R689 are mutated in more than 20 separate cancer cases according to the COSMIC database (Fig. 4). Previous studies have reported that mutations in the WD40 domain prevent interactions between FBXW7 and its known substrates. Recent studies suggest that specific FBXW7 mutations can affect degradation of specific substrates. In fact, FBXW7 D510E retains its ability to target and degrade cyclin E, Mcl-1, and c-Myc, but not NICD [60]. The reason for FBXW7’s D510E lack of NICD degradation is unknown, and FBXW7 D510E shows higher oncogenic activity compared to other FBXW7 mutations [60].

**Table 1:** Gene Family: F-box and WD repeat domain containing (FBXW)

Gene	Mutation Percentage	Tested Samples	Simple Mutations	Fusions	Coding Mutations
FBXW7	2.54	47,844	1216	0	1216
FBXW10	0.58	31,969	184	0	184
BTRC	0.53	32,582	173	0	173
FBXW8	0.47	32,150	151	0	151
FBXW5	0.43	32,062	137	0	137
FBXW12	0.41	32,062	133	0	133
FBXW11	0.39	32,195	124	0	124
FBXW4	0.30	32,062	96	0	96
FBXW2	0.30	31,879	95	0	95
CDRT1	0.14	31,947	45	0	45
FBXW9	0.08	32,062	27	0	27

**Table 2:** SCF-Fbw7 Protein Complex

Gene	Mutation Percentage	Tested Samples	Simple Mutations	Fusions	Coding Mutation
FBXW7	2.54	47,844	1216	0	1216
CUL1	0.71	32,150	228	0	228
SKP1	0.09	32,150	30	0	30
RBX1	0.05	32,150	17	0	17

**Fig. 5:**
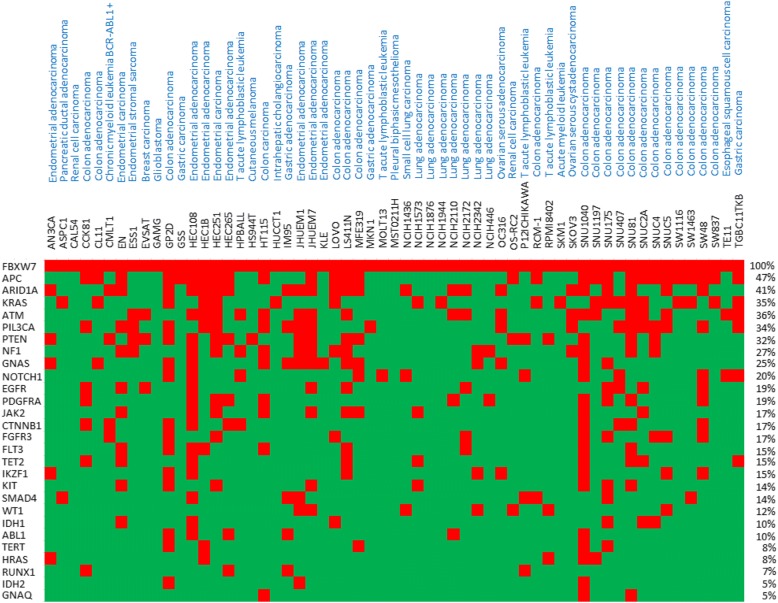
The co-occurrence of FBXW7 mutations with the 50 most frequently mutated genes. The 50 most frequently mutated genes are identified from the COSMIC database. The co-occurrence of FBXW7 mutation and 50 most frequently mutated genes are downloaded from the cBioPortal database. The top panel shows the cancer tissue types and the names of the cancer cell lines with FBXW7 mutations. Red boxes indicate genetic mutations and green boxes represent wild-type gene

To identify potential gene mutation co-occurrences with FBXW7 mutations, we retrieved the 50 most frequently mutated genes from the COSMIC database. We then used the cBioPortal to analyze co-occurrence in the Cancer Cell Line Encyclopedia (CCLE). We found 27 gene mutations that demonstrate significant co-occurrence with FBXW7 mutations (Fig. 5). Among these, as much as 47% of FBXW7 mutant cells also harbor APC mutations. KRAS (Kirsten Rat Sarcoma Viral Proto-Oncogene), ATM, and PTEN were also among the most mutated genes in FBXW7-mutated cancer cells. Whether the combination of FBXW7 mutation along with specific genes can accelerate cancer progression warrants further investigation. FBXW7 dimerization increases binding strength with substrates, which is particularly important for substrates with weak CPDs, like cyclin E. Since FBXW7 mutations are generally heterozygous, how mutations may affect protein function and target degradation may fluctuate [79]. Dimerization enhances the trans-auto-ubiquitination and reduces FBXW7 protein half-life [80]. In contrast to FBXW7 monomers, FBXW7 dimers can bind to multiple CPDs within the target protein and allow for ubiquitination to occur at multiple sites. This has been shown for FBXW7 targets, such as cyclin E. Dimerization also allows for the binding of FBXW7 to suboptimal CPD sequences. As for other dimerizing factors, mutations that do not affect the dimerization domain may exert a dominant negative effect, and hence be more deleterious than mutations resulting in allelic loss [81]. Therefore, it is expected that the functional consequences of FBXW7 mutations upon substrates may be quite variable, and responses to targeted therapies might also be unpredictable [82, 83].

### Post-translational regulation of FBXW7: Dimerization, localization, and auto-ubiquitination

Alterations in FBXW7 cellular distribution can impair its ability to interact with and degrade oncoproteins. Nucleophosmin (NPM) is frequently mutated in acute myelogenous leukemia (AML) and is essential for nucleolar localization and stabilization of FBXW7γ [84]. Loss of NPM destabilizes FBXW7γ and results in c-Myc accumulation [84]. In addition, Protein Kinase C (PKC) phosphorylates FBXW7α at Ser10 and Ser18 [85]. Phosphorylation at Ser10 leads to inhibition of one of FBXW7’s nuclear localization signals (NLS); however, full redistribution of FBXW7α to the cytoplasm requires additional disruption of FBXW7α’s second NLS. PKC may facilitate cellular transformation by inactivating FBXW7α through cytoplasmic sequestration. Finally, the EBP1 (ErbB3 receptor-binding protein) gene encodes two alternatively spliced isoforms: P48 and P42 [86]. P48 forms a negative feedback loop with FBXW7α, whereby FBXW7α degrades P48 in a GSK3-dependent manner, and P48 is able to bind FBXW7α and sequester it in the cytosol. This prevents FBXW7α from degrading its substrates [86]. In contrast, P42 acts as an adaptor to stabilize the interaction between FBXW7 and its substrates, which enhances FBXW7-mediated degradation [86]. The EBP2 (EBNA1-binding protein 2) protein also facilitates different isoforms of FBXW7 nucleolar distribution [87]. Some viral oncoproteins can inactivate FBXW7 function. For example, the large T-antigen of SV40 virus can act as a decoy and target FBXW7γ to the nucleoplasm instead of the nucleolus [88].

FBXW7 function can be regulated through auto-ubiquitination. Peptidyl-prolyl cis-trans isomerase NIMA-interacting 1 (Pin1) has been shown to negatively regulate FBXW7 protein expression [89]. Mechanically, Pin1 interacts with FBXW7 in a phosphorylation-dependent manner and binds to the Thr205-Pro region of FBXW7. This introduces a conformational change in FBXW7, leading to decreased dimerization and increased FBXW7 self-ubiquitination and degradation [89]. Depletion of Pin1 results in up-regulation of FBXW7 and reduced levels of FBXW7 substrates such as Mcl-1, and sensitizes cancer cells to the chemotherapy drug Taxol [89]. Consistently, Sorafenib, which is associated with reduced expression of Pin1, leads to FBXW7 stabilization and destabilization of Mcl-1 [90]. The extracellular signal-regulated kinase (ERK) can also interact with and phosphorylate FBXW7 at Thr205, leading to FBXW7 ubiquitination and proteasome-mediated degradation [91]. The significance of ERK-mediated phosphorylation of FBXW7 was demonstrated by the FBXW7 mutation Thr205A providing resistance to ERK-mediated phosphorylation and suppressed pancreatic cancer cell proliferation and tumorigenesis [91]. In addition to the Thr205 site, polo-like kinase 2 (Plk2) interacts with and phosphorylates FBXW7 on Ser176 (and to a lesser extent Ser25 and Ser349), triggering FBXW7 proteasome degradation [92]. In fact, the oncogenic activity of Plk2 can be reversed by restoring FBXW7 expression [93]. Members of the SCF ubiquitin ligase complexes can also control FBXW7 stability. For example, CSN6, a component of the COP9 signalosome, positively regulate E3 ubiquitin ligases. CNS6 regulates neddylation of Cullin1 (an adaptor for FBXW7 in the SCF ubiquitin ligase complex) and promotes the auto-ubiquitination and degradation of FBXW7 [94]. On the other hand, mono-allelic deletion of CSN6 decreases Cullin1 neddylation, stabilizes FBXW7, and compromises lymphomagenesis in an Eμ-Myc mouse model [94]. Deletion of Glomulin (Glmn), a member of the SCF complex that interacts with Rbx1, reduces FBXW7 expression by stimulating Rbx1-mediated ubiquitination of FBXW7 [95]. In all, there are over 300 different interacting partners for FBXW7 that are described in the BioGRID database [96]. Among them, some have been shown to affect FBXW7 stability. For example, the family with sequence similarity 83, member D (FAM83D) interacts with FBXW7 and induces its proteasome-dependent degradation. FAM83D-mediated FBXW7 down-regulation is associated with an increase in the expression of FBXW7 substrates, such as c-Myc, mTOR and c-Jun, in breast cancer; and FAM83D is frequently deregulated in breast and colon cancer [97, 98]. The auto-ubiquitination and stability of FBXW7 can be reversed by the deubiquitinase (DUB) Usp28 [99]. Although single allele deletion of Usp28 allows FBXW7-mediated substrate degradation, it has little effect on FBXW7 stability. However, Usp28 null cells show Pin1-dependent, FBXW7 degradation and accumulation of FBXW7 substrates [99].

### Disruption of FBXW7-substrate interactions

The FBXW7 WD40 domain forms a β-propeller structure involved in substrate binding to the CPD region of the targeted protein. Mutations in specific CPD residues can disrupt FBXW7-mediated targeting and degradation. Hotspot mutations within the CPD motif of c-Myc are detected at residue Thr58 in Burkitt’s lymphoma patient samples. Mechanistically, mutated Thr58 can no longer be phosphorylated by GSK3, resulting in increased c-Myc half-life and higher oncogenic potential [100]. Pro57 is also highly mutated in Burkitt’s lymphoma cells and is required for Thr58 phosphorylation. COSMIC database analyses revealed that mutation of Pro2514 within the Notch1 CPD (FLTPSPES) accounts for as much as 14.63% of the total Notch1 mutations found across all human cancers (Fig. 1). Likewise, COSMIC database analyses revealed that a Pro59 mutation within the c-Myc CPD accounted for more than 9% of all c-Myc mutations found in human cancer (Fig. 1). Proline mutations are predicted to have serious consequences for protein conformation and the role of these mutations in preventing Notch1 or c-Myc binding to FBXW7 warrants further analyses. In the absence of genetic mutations, alterations in signaling pathways controlling phosphorylation of FBXW7 targets play an important role. Along these lines, because GSK3β-mediated phosphorylation is required for degradation of c-Myc [12, 101], cyclin E [4] and MCL1 [46], any signaling pathway that affects GSK3β activity can have a profound impact on FBXW7’s ability to perform its tumor suppressor activities. In addition, de-regulation of GSK3 upstream modulators also leads to altered FBXW7 functions. As stated earlier, this is the case for AURKA regulation by PTEN. Loss of PTEN leads to alterations in the PI3K/AKT/GSK3 pathway that stabilizes AURKA [58]. In addition to GSK3β, other kinases are also important in FBXW7 functions. For instance, CDK5-mediated EZH2 phosphorylation is essential for FBXW7-mediated EZH2 degradation [77] and CDK8 is required for Notch1 degradation [35]. In contrast to reducing FBXW7-substrate binding, some proteins are reported to increase FBXW7-substrate binding, hence reducing oncoprotein expression. For instance, amplified in breast cancer 1 (AIB1) is a transcriptional coactivator that activates the transcription of nuclear receptors and other transcription factors [102]. PTEN has been shown to interact with both AIB1 and FBXW7 [102]. This interaction brought AIB1 and FBXW7α together and accelerated AIB1 degradation [102]. Furthermore, Alpha-synuclein (SNCA) promotes the degradation of the NICD domain via directly interacting with FBXW7 and promoting the interaction between Notch1 and FBXW7 [103]. Finally, a direct competition mechanism and displacement of FBXW7 targets by viral proteins has been reported. For example, Kaposi’s sarcoma-associated herpesvirus (KSHV) latency-associated nuclear antigen (LANA) can form a complex with FBXW7. The LANA/FBXW7 complex inhibits NICD ubiquitination and degradation by competing with NICD for binding to FBXW7 [104]. Hence, KSHV-infected tumor cells increase cell proliferation by reducing FBXW7-mediated Notch degradation. Merkel cell polyomavirus (MCV) contains small T (ST) and large T (LT) viral oncoproteins that are involved in aggressive human skin cancer [105]. While the LT viral oncoprotein is a substrate of FBXW7, the ST viral oncoprotein contains an LT-stabilization domain (LSD) inhibiting FBXW7 interaction with the LT viral oncoprotein [105].

## Clinical significance of FBXW7 loss of function in human cancers

### Acute lymphoblastic leukemia

Conditional knock-out of FBXW7 in hematopoietic cells or T cells is sufficient to cause T-ALL or thymic lymphoma by increasing Notch1 and c-Myc expression [7, 106]. However, FBXW7 hotspot mutation knock-in mice (FBXW7mut/+) do not develop spontaneous leukemia, demonstrating a subtle difference between FBXW7 missense mutations and homologous deletion. When combining FBXW7 deletion/mutation with p53 suppression, loss of PTEN, or active Notch, an FBXW7 deletion/mutation enhances tumorigenesis [7, 16, 58]. Notch and NF-κB can bind to the miR-223 promoter and activate miR-223 expression [107]. Studies show that Notch-mediated activation of miR-223 suppresses FBXW7 in T-ALL; and miR-223 and FBXW7 expression are inversely correlated in T-ALL patient-derived xenografts [107]. The oncogenic transcription factor, T-cell acute lymphocytic leukemia 1 (TAL1/SCL), is aberrantly expressed in more than 60% of T-ALL patients [108]. TAL1 binds to the miR-223 promoter and increases miR-223 expression. Silencing TAL1 increases expression of FBXW7 and lowers expression of FBXW7 substrates, such as c-Myc, Notch1 and cyclin E [108]. Therefore, it appears that miR-223 plays an essential role in FBXW7 activities. In addition, several studies demonstrate a role for miR-27a in ALL. MiR-27a is up-regulated in pediatric B-ALL and its expression is inversely correlated with FBXW7 expression and disease progression [69].

Results from multiple ALL clinical trials suggests an important role for FBXW7 in patient survival. In 47 patients with pediatric T-ALL, FBXW7 mutations are not associated with treatment outcome [109]. This is equivalent to the 88 T-ALL patients who were treated on the UKALLXII/ECOG E2993 protocol, a chemotherapy regime specific to newly diagnosed adult ALL patients [110]. However, in T-ALL patients treated in either the Lymphoblastic Acute Leukemia in Adults-94 trials (LALA-94) (*n* = 87) or the Group for Research on Adult Acute Lymphoblastic Leukemia-2003 trials (GRAALL-2003) (*n* = 54), Notch1/FBXW7-mutated patients have better overall survival and event-free survival compared with other patients, although there is no correlation between Notch1/FBXW7 mutation and clinical biologic features [111]. Glucocorticoid receptor alpha (GRα) may better explain the clinical outcomes in patients with FBXW7 mutations. FBXW7 is known to ubiquitinate and degrade GRα. Inhibition of FBXW7 leads to increased glucocorticoid sensitivity, but not other chemotherapy drugs for T-ALL [112].

### Colorectal cancer

FBXW7 mRNA expression is reported to be significantly reduced in colorectal cancer (CRC) [113]. Using a comparative genomic hybridization (CGH) array, over 20% of CRC patients (*n* = 130) have copy number loss of FBXW7, which correlate with disease progression [113]. Among all human cancers, large intestine cancer harbors the second most frequent FBXW7 mutations (7.73%). Metastatic CRC (mCRC) patients with FBXW7 missense mutations show shorter overall survival when compared with patients with wild-type FBXW7 [114]. Surprisingly, younger adult colorectal cancer patients have a higher FBXW7 mutation frequency compared to patients diagnosed at an older age [115]. FBXW7 and SMAD4 (SMAD, Mothers Against DPP Homolog 4) mutations are prevalent in CRC patients resistant to anti-epidermal growth factor receptor (EGFR) immunotherapy treatment (monoclonal antibodies, Cetuximab or Panitumumab) [116]. In addition, loss of FBXW7 is associated with drug resistance to Oxaliplatin, a drug commonly used to manage mCRC [117].

Analyses of normal colonic epithelial cells against primary and metastatic tumor cells from matched samples show de novo mutations of FBXW7 in synchronous and/or metachronous liver and/or lung metastases [118]. Furthermore, de novo mutations of FBXW7 occur more frequently in metachronous lung metastases compared with synchronous lung metastases [118]. Similarly, the somatic genetic evolution of colorectal adenocarcinoma (COAD) show that APC and TP53 mutations preferentially happen early, while alterations in FBXW7 and PTEN preferentially occur as a late event [119]. Although FBXW7 mutations are found in 14.3% of colorectal cancer, no one clinical, pathological or demographic feature is representative of the patients with FBXW7 mutations [120]. In a screen of 96 CRC patients, 10.8% harbored FBXW7 mutations that are localized in the substrate-binding arginine residues of the β-propeller blade of FBXW7 (R278, R465, R479, and R505). Importantly, of the 96 CRC patients screened, 8.6% had co-mutations in FBXW7 and in the oncogene KRAS. This is similar to a study of patients with FBXW7 mutations and CRC, of which 86% also harbor KRAS mutations [120]. Despite the prevalence of FBXW7 mutations, there is no difference between CRC patients with and without an FBXW7 mutation in the five-year overall survival [121, 122]. In 822 patients from the VICTOR trial of stage II/III colorectal cancer, mutation of FBXW7 is not associated with disease-free survival [122].

### Esophageal and gastric cancers

FBXW7 mRNA expression is reduced in esophageal squamous cell carcinoma (ESCC) [123]. FBXW7 is also associated with muscle-invasive tumor cases (T2–4), lymphatic-invasive tumor cases, and stage II-IV cases [124]. ESCC patients with loss of FBXW7 copy number show lower FBXW7 expression and poorer clinical prognosis [123]. Loss of heterozygosity (LOH) of FBXW7 is detected in more than 30% of early-onset gastric cancers (EOGC) and associates with loss of FBXW7 expression in more than 25% of EOGC [125]. In addition, it is reported that the expression of FBXW7 mRNA is reduced in paired tumor and non-tumor tissues in gastric adenocarcinoma patients [126]. Similarly, single allele deletion of FBXW7 is observed in 45.5% of gastric tumors, and coordinately, c-Myc amplification is found in 51.5% of gastric tumor samples [127]. Importantly, immunohistochemistry shows that down-regulation of FBXW7 protein expression is correlated with poor prognosis and adjuvant chemotherapy response [126]. Aberrant FBXW7 mRNA expression is correlated with lymph node metastasis and tumor stage III-IV [127]. Additional studies show that reduced FBXW7 expression is correlated with lymph node metastasis, tumor size, and poor prognosis in primary gastric cancer [128]. This is mainly due to mutations in p53, which lead to reductions in FBXW7 transcripts [128]. Loss of FBXW7 is not associated with ploidy status [125]. In gastric carcinoma, inhibition of miR-25 induces cell apoptosis through up-regulation of FBXW7 and down-regulation of FBXW7 substrates, such as cyclin E and c-Myc [129]. Additionally, miR-223 is significantly up-regulated in gastric cancer, pancreatic ductal adenocarcinoma, and ESCC [130–132]. ESCC tissues having lower FBXW7 and higher miR-223 expression show poorer overall survival and lower 5-year survival [131]. In gastric cancer, resistance to the DNA damaging drug cisplatin is the most common cause of chemotherapy failure. miR-223 is found to promote cisplatin resistance of human gastric cancer cells by targeting FBXW7 [133].

### Hepatocellular carcinoma

FBXW7 expression is also reduced in hepatocellular carcinoma (HCC) tissues [134, 135]. HCC patients with high histological grade and advanced tumor-node-metastasis stage show lower FBXW7 protein expression [135]. Furthermore, FBXW7 protein expression in HCC tissues is inversely correlated with FBXW7 substrates c-Myc and cyclin E [135]. FBXW7 expression is reduced in HCC tissues compared to normal tissue and correlated with tumor differentiation, the incidence of portal or hepatic venous invasion, and metastasis [136]. Furthermore, lower FBXW7 expression is associated with poor clinical pathology features, including large tumor size, high pathological grading, and advanced TNM stage. Importantly, higher FBXW7 mRNA expression is associated with a better five-year overall survival and a longer disease-free survival rate, and expression of FBXW7 could be used as an independent risk factor for HCC recurrence [134, 137]. Similarly, in intrahepatic cholangiocarcinoma (IHCC), FBXW7 expression is associated with disease stage, lymph node metastasis, three-year survival rates, and overall and disease-free survival [138]. Two miRNAs, miR-27b and miR-92a, are overexpressed and can target FBXW7 in HCC. miR-27b inhibits FBXW7 expression and is associated with poor clinical outcome [139], while miR-92a is inversely correlated with FBXW7 expression, increased cell proliferation, and inhibited apoptosis of HCC [140]. The lncRNA CASC2 acts as a sponge for miR-367 in HCC samples [66]. CASC2 enhances FBXW7 expression by inhibiting miR-367 down-regulation of FBXW7. HCC tissues show considerable repression of CASC2, which correlates with metastasis symptoms and poor prognosis.

### Non-small cell lung cancer

FBXW7 expression is down-regulated in non-small cell lung cancer (NSCLC) and patients with low FBXW7 expression have more aggressive cancer and a shorter survival time [141]. In the clinic, EGFR inhibitor-resistant NSCLC specimens show down-regulation of FBXW7, which is associated with up-regulation of Mcl-1 expression [142]. Reactivation of FBXW7 reduces Mcl-1 expression and sensitizes resistant cells to targeted therapy [142]. In NSCLC, loss of FBXW7 increased the sensitivity of a class I-specific histone deacetylase (HDAC) inhibitor, MS-275 [141]. Tissues with low expression of FBXW7 display increased Mcl-1 and TOP2A (DNA topoisomerase II), an additional FBXW7 target. EGFR-tyrosine kinase inhibitors, such as Erlotinib, have shown some success with EGFR+ or EGFR mutated cancers, however resistance can occur over time. Recently, the miR-223/FBXW7 axis has also been shown to be involved in Erlotinib drug resistance in NSCLC cells [143]. Resistance partially occurs through up-regulation of miR-223, which down-regulates FBXW7. This effect can be reversed by inhibitors targeting either miR-223, AKT or the Notch pathway [143]. FBXW7 is also found to be targeted by miR-25 and miR-367 in NSCLC cells [144, 145]. In addition, p53 mutations can lead to increased expression of miR-25 [146] and overexpression of miR-25 increases NSCLC cell proliferation and migration by targeting FBXW7 [144]. LncRNA MALAT1 is known to reduce metastasis and could serve as a biomarker to predict lung cancer prognosis [67, 147, 148]. Consistent with the fact that FBXW7 is a direct target of miR-155, MALAT1 was shown to increase expression of FBXW7 by sequestering miR-155 [67]. However, levels of MALAT1 are elevated in NSCLC, which suggests MALAT1 and/or miR-155 do not play a significant role in FBXW7 regulation and implies that the MALAT1/miR-155/FBXW7 axis may have a tumor suppressor role in lung cancer. This is in contrast to its oncogenic role in glioma patient samples, where tissue samples show reductions in MALAT1 levels compared to non-cancerous brain tissue [67]. FBXW7 is identified as a genuine target of miR-155 in glioma cells. The different roles for MALAT1 may be due to differential expression during different stages of disease or different cell-type specific MALAT1 targets. In fact, MALAT1 is also found to target the miR-124a/STAT3 axis in NSCLC [147].

### Breast cancer

FBXW7 mRNA expression is lower in breast cancer compared with normal tissues [149]. In addition, one report finds that 80% of breast cancer cell lines harbor alternatively spliced isoforms of FBXW7 [150]. These alternatively spliced forms allow for higher translational efficiency and increased protein expression of FBXW7. Therefore, deregulation of specific alternatively spliced isoforms of FBXW7 may explain the down-regulation of FBXW7 in some human tumors. Mutations in FBXW7 are relatively rare in breast cancer patients. Approximately 1% of breast cancer patients harbor point mutations in FBXW7, whereas 30% of breast cancer lines and patients have deletions in the FBXW7 chromosome 4q31 [149, 151, 152]. When analyzing different breast cancer molecular subtypes (normal-like, luminal A, luminal B, ERBB2 (Erb-B2 Receptor Tyrosine Kinase 2), and basal), ERBB2 and basal tumors show significantly lower FBXW7 expression compared to normal-like tumors [149]. In addition, luminal A and B tumors show the lowest FBXW7 mRNA expression in breast cancer subtypes [149]. Similarly, breast cancer patients with high histological grade and hormone receptor-negative tumors show lower expression of FBXW7 [153]. Lower expression of FBXW7 is associated with a high Ki-67 labeling index and positive cyclin E protein expression, both markers of proliferation [153]. Disease-free survival is greater in breast cancer patients demonstrating the highest gene expression of FBXW7 [149].

The association between FBXW7 expression and overall survival is complicated. In ER negative, ERBB2, and basal subtype tumors, patients with higher FBXW7 expression increased their overall survival [149]. However, higher FBXW7 is correlated with poor overall survival in the normal-like subtype [149]. Since FBXW7 can target different substrates in a cell-context dependent manner, further studies are needed to identify the role of FBXW7 in different subtypes of breast cancer. Interestingly, an independent study found that methylation of FBXW7β is associated with favorable prognosis [74]. Additionally, analysis of FBXW7 mutational hotspots in various cancers, including primary breast cancer, show deamination of 5′-methylcytosine within a CG dinucleotide at R479 [5]. The cytosine at this hotspot is methylated, which could lead to inactivation of FBXW7 in breast and other cancerous tissue. Reports have shown that in breast cancer tissue samples miR-32 is overexpressed and inversely correlates with FBXW7 [72]. Overexpression of miR-32 reduces apoptosis and increases cell proliferation and migration by targeting FBXW7 [72]. A similar expression profile is seen for miR-182 in breast cancer tissue, where miR-182 regulates invasiveness, proliferation, and cell cycle progression [154]. miR-182 expressing cells are highly sensitive to hypoxia with up-regulation of HIF-1α (hypoxia inducible factor-1) and VEGF-A (vascular endothelial growth factor A) [154]. Importantly, overexpression of FBXW7 reverses the increases in HIF-1α and VEGF-A expression [154]. Preliminary reports may also demonstrate a role for miR-367a in reducing FBXW7 expression in breast cancer samples.

## Conclusions

FBXW7 is an essential tumor suppressor and is frequently inactivated in human cancer cells (Table 3). This may offer opportunities to selectively target FBXW7 mutant cells through synthetic-lethal methods or oncogene dependence. This is possible by targeting genes that are selectively essential in cell lines carrying particular driver mutations, but not normal cells with wild-type FBXW7. In support of this model, human breast cancer cells with loss of FBXW7 are hypersensitive to rapamycin [50]. Therefore, inactivation of FBXW7 can be targeted by an mTOR inhibitor to reverse the oncogenic effect of an FBXW7 mutation [50]. As another example, FBXW7 has been demonstrated to target GRα in T-ALL. Reduced FBXW7 expression or function increased the glucocorticoid sensitivity in T-ALL, but not the sensitivity to other chemotherapy drugs [112]. Thus, glucocorticoid treatment can provide a therapeutic window to selectively block FBXW7 mutant cells and spare normal FBXW7 wild-type cells. In addition, down-regulation of GSK3β reduces c-Myc T58 phosphorylation, which impairs FBXW7-mediated c-Myc degradation. The accumulation of c-Myc increases the expression of TRAIL5 (TNF-related apoptosis-inducing ligand death receptor 5), which increases TRAILR5 (death receptor 5)-induced apoptosis both in vitro and in vivo [155]. This may be used to target c-Myc-overexpressing cells in FBXW7-deficient cell lines. Similarly, the histone deacetylase (HDAC) inhibitor Vorinostat induces apoptosis through down-regulation of Mcl-1 and up-regulation of the pro-apoptotic proteins Noxa (PMA-Induced Protein 1) and Bim (BCL2 Like 11) [49]. Mutation of FBXW7 in squamous cell carcinoma (SCC) increases Mcl-1and Bim expression, which leads to resistance to standard chemotherapy but increases susceptibility to HDAC inhibitors [49]. When combining HDAC inhibitors with BH3-mimetic ABT-737, cancer cells show hypersensitivity to the treatment [49]. Finally, human colorectal cells deficient in FBXW7 are dependent on spindle assembly checkpoint proteins BUBR1, BUB1, and MPS1 [156]. Reducing BUBR1 in cells lacking FBXW7 leads to cell aneuploidy and up-regulation of p53 expression [156]. While most studies focus on the cell-autonomous function of FBXW7 in tumor cells, it has also been shown that FBXW7 affects the tumor microenvironment and inhibits tumor metastasis [157]. Specifically, knockout of FBXW7 in mouse bone marrow-derived stromal cells increases Notch expression and then activates chemokine CCL2 (C-C motif chemokine ligand 2) expression [157]. Increased CCL2 in serum recruits both monocytic myeloid-derived suppressor cells and macrophages to the tumor site, thereby increasing tumor metastasis [157]. Importantly, the effect of FBXW7 depletion can be reversed by a CCL2 receptor antagonist, which provides a therapeutic strategy to target FBXW7-deficient cells in clinics [157].

**Table 3:** Clinical Significance of FBXW7 Loss-of-function

Cancer	Association between FBXW7 loss-of-function and patient clinical outcomes
B-ALL	Pediatric B-ALL: FBXW7 is inversely correlated with disease progression.
T-ALL	Overall, FBXW7 is important in disease survival and is linked to specific drug/chemotherapy regime in T-ALL.
	Mutation(s) in FBXW7 are not associated with treatment outcomes in newly diagnosed adult T-ALL (UKALLXII/ECOG E2993 regime) or pediatric T-ALL. However, mutation(s) in FBXW7 are linked to better overall survival and event-free survival in LALA-94 and GRAALL-2003 treatment groups.
	Activating mutations in Notch (Notch or FBXW7) in pediatric T-ALL lead to better overall response to chemotherapy, but overall patient outcomes differ.
Colorectal Cancer (CRC)	FBXW7 mutation(s) do not correlate with 5 year overall survival or disease-free survival in CRC; however, distinct mutations had better overall survival than others.
	In metastatic CRC patients (mCRC) missense mutation(s) or loss of FBXW7, demonstrate shorter overall survival and drug resistance to Oxaliplatin, respectively.
	FBXW7 mutation(s) show resistance to anti-epidermal growth factor receptor (EGFR) immunotherapy treatment (ie. Cetuximab or Panitumumab).
Esophageal and Gastric Cancers (ESCC)	FBXW7 is associated with muscle-invasive tumor cases (T2–4), lymphatic-invasive tumor cases, and stage II-IV cases.
	Copy number loss of FBXW7 is associated with poorer clinical outcome; while Loss of FBXW7 protein expression is correlated with poor prognosis and adjuvant chemotherapy response.
	Aberrant FBXW7 mRNA expression is correlated with lymph node metastasis and tumor stage III-IV.
	Reduced FBXW7 expression is correlated with lymph node metastasis, tumor size, and poor prognosis in primary gastric cancer.
	Lower FBXW7 in ESCC tissue show poorer overall survival and lower 5-year survival.
	Targeting of FBXW7 can lead to Cisplatin resistance in human gastric cancer cells.
Hepatocellular Carcinoma (HCC) and intrahepatic cholangiocarcinoma (IHCC)	Lower FBXW7 expression correlates in HCC patients with higher histological grade and advanced tumor-node-metastasis.
	In HCC patients, FBXW7 correlates with tumor differentiation, the incidence of portal or hepatic venous invasion, metastasis, and poorer clinical pathologic features (including large tumor size, high pathological grading, and advanced TNM stage).
	Low expression of FBXW7 in both tumor and non-tumor tissue is an independent risk factor for HCC recurrence in patients after hepatectomy.
	Reduced FBXW7 expression correlates with tumor progression and poor prognosis in IHCC.
Non-small cell lung cancer (NSCLC)	Lower FBXW7 expression is associated with more aggressive cancer and shorter survival time in NSCLC.
	FBXW7 down-regulation is associated with EGFR inhibitor-resistance in NSCLC.
Ovarian Carcinoma	Lower FBXW7 expression in ovarian carcinomas, compared to borderline or benign tumors.
	FBXW7 expression levels were lowest in serious carcinomas, followed by endometrioid carcinomas, clear cell carcinomas, and mucinous carcinomas.
Glioblastoma	Lower FBXW7 expression in patients with grade IV glioma.
	FBXW7 serves as a prognostic marker in glioblastoma; with levels of FBXW7 correlating with survival.
	FBXW7 expression is inversely correlated with glioma histology and positively correlated with patient survival time.
Breast Cancer	Higher FBXW7 gene expression correlates with increased disease-free survival in breast cancer patients, especially ER negative and basal subtypes.
	Higher FBXW7 expression was associated with increased overall survival in patients with ER negative, ERBB2, and basal subtype tumors.
	Higher FBXW7 expression was associated with decreased overall survival in patients with normal-like subtype.
	Overall, FBXW7β methylation is associated with favorable prognosis and poorly differentiated tumors in breast cancer.
Pancreatic Cancer	Low expression of FBXW7 is an independent poor prognostic factor for pancreatic cancer; low FBXW7 correlated with overall, cancer-specific, and relapse-free survival rates.
	Levels of FBXW7 expression are associated with sensitivity to gemcitabine treatment.

Although a lot of progress has been made regarding the regulation and function of the tumor suppressor FBXW7 in normal and tumoral cells, many questions still remain open-ended. For instance, the possibility that mutated forms of FBXW7 have oncogenic effects offers an opportunity for targeted therapies. Along these lines, recent studies suggest that, unlike the hot spot arginine mutations (R465, R479 and R505), some mutations may differentially affect degradation of some FBXW7 targets. A better understanding of the conformational changes responsible for these phenotypes may also allow for the design of small inhibitors that selectively affect specific downstream signaling pathways and/or to alter FBXW7 substrate selection. Since FBXW7 mutations are frequently heterozygote, it is also important to understand the contribution of FBXW7 monomers as well as dimers within a both mutated or a single mutated unit.

## Abbreviations

ALL: Acute lymphoblastic leukemia
AML: Acute myelogenous leukemia
APC/C: Anaphase Promoting Complex/Cyclosome
API-1: AKT inhibitor
ATL: Adult T-cell leukemia
ATM: ATM Serine/Threonine Kinase
AURKA: Aurora kinase A
B-ALL: B-cell acute lymphoblastic leukemia
Bim: BCL2 Like 11
CCL2: C-C motif chemokine ligand 2
CCLE: Cancer Cell Line Encyclopedia
CML: Chronic myelogenous leukemia
COAD: Colorectal adenocarcinoma
COSMIC: Catalogue of Somatic Mutations in Cancer
CPD: CDC4 phosphodegron
CRC: Colorectal cancer
Cul-1: Cullin/Cdc53
cyclin E/CDK2: Cyclin E/cyclin-dependent kinase Cdk2
DNMT1: DNA methyltransferase 1
DSBs: Double DNA strand breaks
EBP1: ErbB3 receptor-binding protein
EBP2: EBNA1-binding protein 2
EGFR: Epidermal growth factor receptor
EOGC: Early-onset gastric cancers
ERBB2: Erb-B2 Receptor Tyrosine Kinase 2
ERK: Extracellular signal-regulated kinase
ESCC: Esophageal squamous cell carcinoma
EZH2: Enhancer of zeste homolog 2 polycomb repressive complex 2
FAM83D: Family with sequence similarity 83, member D
FBXW7: F-box and WD repeat domain containing 7
Glmn: Glomulin
GRAALL-2003: Group for Research on Adult Acute Lymphoblastic Leukemia-2003 trials
GRα: Glucocorticoid receptor alpha
GSK3β: Glycogen synthase kinase 3 beta
HCC: Hepatocellular carcinoma
HDAC: Histone deacetylase
HIF-1α: Hypoxia inducible factor-1
HTLV-I: Human T-cell leukemia virus
IHCC: Intrahepatic cholangiocarcinoma
ILK: Integrin-linked kinase
JNKs: Jun N-terminal kinases
KRAS: Kirsten Rat Sarcoma Viral Proto-Oncogene
KSHV: Kaposi’s sarcoma-associated herpesvirus
LALA-94: Lymphoblastic Acute Leukemia in Adults-94 trials
LIC: Leukemia-initiating cell
lncRNA: long non-coding RNA
LOH: Loss of heterozygosity
LSD: LT-stabilization domain
MAPK: Mitogen-activated protein kinase
Mcl-1: Myeloid cell leukemia-1
MCV: Merkel cell polyomavirus
miRNA: microRNA
mTOR2: mTOR complex 2
NHEJ: Non-homologous end joining
NICD: Notch nuclear intracellular domain
NLS: Nuclear localization signals
Notch: Notch (Drosophila) Homolog 1 Translocation-Associated)
Noxa: PMA-Induced Protein 1
NPM: Nucleophosmin
NSCLC: Non-small cell lung cancer
PIKK: Phosphoinositide 3-kinase-related kinase
Pin1: Peptidyl-prolyl cis-trans isomerase NIMA-interacting 1
PKC: Protein Kinase C
Plk2: Polo-like kinase 2
PS: Presenilin
PTEN: Phosphatase and tensin homologue deleted on chromosome 10
Rack1: Receptor for activated C-kinase 1
RCC: Renal cell carcinoma
RICTOR: Rapamycin-insensitive companion of mammalian target of rapamycin
SAC: Spindle assembly checkpoint
SCC: Squamous cell carcinoma
SCF/β-TrCP: Skp1-Cdc53/Cullin-F-box-protein complex
SMAD4: Mothers Against DPP Homolog 4
SNCA: Alpha-synuclein
TAL1/SCL: Squamous cell carcinoma
TOP2A: DNA topoisomerase II
TRAIL5: TNF-related apoptosis-inducing ligand death receptor 5
UbcH5: Ubiquitin Conjugating Enzyme E2 D1
UPS: Ubiquitin-proteasome system
USP: Ubiquitin-specific proteases
VEGF-A: Vascular endothelial growth factor A
WD40: Tryptophan-aspartic acid motif
XRCC4: DNA repair protein X-Ray Repair Cross Complementing 4
